# Epratuzumab inhibits the production of the proinflammatory cytokines IL-6 and TNF-α, but not the regulatory cytokine IL-10, by B cells from healthy donors and SLE patients

**DOI:** 10.1186/s13075-015-0686-2

**Published:** 2015-07-17

**Authors:** Vanessa Fleischer, Julia Sieber, Sarah J. Fleischer, Anthony Shock, Guido Heine, Capucine Daridon, Thomas Dörner

**Affiliations:** Department Medicine/Rheumatology and Clinical Immunology, Charité University Medicine Berlin, CC12, Charité Berlin, Charitéplatz 01, 10098 Berlin, Germany; German Rheumatism Research Center Berlin (DRFZ), Leibniz Institute, Berlin, Germany; UCB Celltech, Slough, UK

## Abstract

**Introduction:**

Cytokines produced by B cells are believed to play important roles in autoimmune diseases. CD22 targeting by epratuzumab has been demonstrated to inhibit phosphorylation of B cell receptor (BCR) downstream signaling in B cells. It has been shown that other sialoadhesin molecules related to CD22 have immunoregulatory functions; therefore, in the present study, we addressed the role of epratuzumab on the production of key cytokines by B cells of patients with systemic lupus erythematosus (SLE) and of healthy donors (HD).

**Methods:**

Peripheral blood B cells were purified and activated by BCR with or without Toll-like receptor 9 (TLR9) stimulation in the presence or absence of epratuzumab. Cytokine production by B cells (interleukin [IL]-6, tumor necrosis factor [TNF]-α and IL-10) in the supernatant and the induction of IL-10^+^ B cells from patients with SLE and HD were analyzed.

**Results:**

The secretion of the proinflammatory cytokines TNF-α and IL-6 by anti-BCR and BCR- and/or TLR9-activated B cells from HD and patients with SLE was inhibited by epratuzumab. In contrast, the production of IL-10 by B cells was not affected by epratuzumab under either stimulation condition. Consistently, the induction of IL-10–producing B cells in culture was not affected by epratuzumab.

**Conclusions:**

Epratuzumab, by targeting CD22, was able to inhibit the production of the proinflammatory cytokines IL-6 and TNF-α by B cells, in contrast to IL-10, *in vitro*. These data suggest that targeting CD22 alters the balance between proinflammatory cytokines (TNF-α, IL-6) and the regulatory cytokine IL-10 as another B cell effector mechanism.

## Introduction

Systemic lupus erythematosus (SLE) is a heterogeneous autoimmune disease with a breakdown of self-tolerance that leads to various immune abnormalities, including the production of autoantibodies to double-stranded DNA and other nuclear antigens, deposition of immune complexes in various organs, and B cell disturbances suggesting a key role for B cells in the pathogenesis of this disease [1, 2].

Epratuzumab, a humanized immunoglobulin G1 (IgG1) monoclonal antibody (mAb) that targets the B cell surface molecule CD22, is currently being tested in clinical trials for the treatment of SLE, and it has been shown to modulate the activation of B cells. In this context, *in vitro* mechanism-of-action studies have shown that epratuzumab binding to CD22 on B cells leads to rapid internalization of the antibody–CD22 complex [3], phosphorylation of immunoreceptor tyrosine–based inhibitory motifs on the CD22 intracellular tail [3], diminished proliferation of isolated B cells from patients with SLE [4], and modification of migration of B cells [5]. Recently, we demonstrated that this anti-CD22 antibody is able to inhibit B cell receptor (BCR) signaling in human B cells [6]. However, to date, whether other B cell functions, such as cytokine production, can also be modulated by epratuzumab has not been reported.

CD22 exclusively expressed by B cells is a member of the sialic acid–binding immunoglobulin-type lectin (Siglec) family, proteins known to modulate a wide range of immune functions on dendritic cells (DCs), macrophages and, in the case of CD22 (Siglec-2), on B cells [7]. In this regard, *cis* signaling of certain Siglec family members is known to regulate the balance of proinflammatory cytokines and the regulatory cytokine interleukin (IL)-10 in DCs and macrophages [7]. Because cytokines produced by B cells following BCR and/or Toll-like receptor (TLR) stimulation have been described as playing an important role in autoimmune diseases [8], and because epratuzumab is able to partially inhibit BCR responses [6], in the present study we analyzed whether the antibody also has the capacity to modulate *in vitro* the cytokine production (IL-6, tumor necrosis factor [TNF]-α and IL-10) by B cells from patients with SLE compared with healthy donors (HD) upon BCR cross-linking alone or in combination with TLR9 stimulation. The latter appears to be involved in autoimmune B cell activation in a T cell–independent manner, allowing us to mimic autoimmune B cell–intrinsic TLR signaling.

## Methods

### Patients and controls

In the studies investigating cytokine production, peripheral blood was collected from 13 patients with SLE (12 females and 1 male) with a mean age of 30.6 ± 8.8 years and with a median Systemic Lupus Erythematosus Disease Activity Index (SLEDAI) score of 6 (range: 4–15) and 9 HD (8 females and 1 male) with a mean age of 33.4 ± 11.5 years. For the activation analysis and IL-10 production of B cells using flow cytometry (FC), peripheral blood was collected from six female patients with SLE with a mean age of 38.8 ± 12.9 years and a median SLEDAI score of 6 (range: 5–18). Ten HD (8 females and 2 males) with a mean age of 32.9 ± 11.1 years served as controls. The study was approved by the ethics committee at the Charité-Universitätsmedizin Berlin, and written consent was obtained from all donors. All patients met the revised American College of Rheumatology classification criteria for SLE [9]. Disease activity was assessed using the Safety of Estrogens in Lupus Erythematosus National Assessment–SLEDAI score [10].

### Peripheral blood mononuclear cells and B cell purification

Peripheral blood mononuclear cells (PBMCs) were isolated by density gradient centrifugation as previously described [11]. B cells were negatively purified magnetically (B Cell Isolation Kit II; Miltenyi Biotec, Bergisch Gladbach, Germany) according to the manufacturer’s instructions. B cells were analyzed regarding their purity to minimize the contamination by other cytokine-producing cells.

### B cell purity

A total of 100,000 purified B cells were stained with antibodies against CD14-PacB (M5E2), CD3-PacB (UCHT1) and CD19 PE-Cy7 (SJ25C1) (all from BD Biosciences, San Jose, CA, USA) for 15 minutes at 4 °C. Afterward, 4′,6-diamidino-2-phenylindole (DAPI) was added to the stained cells (dead cell staining) and analyzed by FC using a FACSCanto II flow cytometer (BD Biosciences). Data were evaluated using FlowJo software (version 7; Tree Star, Ashland, OR, USA). The total B cell (CD3^−^CD14^−^DAPI^−^CD19^+^) purity was 98.5 ± 2.2 % in almost all samples (mean ± standard deviation), with the exception of two cases with 8.2 % and 5.6 % non–B cells (CD3^+^ cells, CD14^+^ cells or cell debris). These two samples were outliers but did not show any results substantially different from the remaining samples. Additional analyses also did not show any relationship between cytokine levels in the supernatants of TNF-α, IL-6 or IL-10 and the frequency of non-B cells of these samples, including any relationship of the impact of epratuzumab and the frequency of non-B cells after purification (data not shown).

### B cell *in vitro* stimulation

Cells were resuspended in RPMI 1640 GlutaMAX medium (Life Technologies, Darmstadt, Germany) supplemented with 10 % fetal calf serum (Lonza, Cologne, Germany), 5 % penicillin-streptomycin and 0.05 mM 2-mercaptoethanol (Gibco; Life Technologies) at 1.1 × 10^6^ purified B cells/ml or 1.1 × 10^7^ PBMCs/ml. Cells (90 μl) were seeded, pretreated with 10 μg/ml F(ab′)_2_ epratuzumab (provided by UCB Celltech, Slough, UK) for 15 minutes and stimulated with 2.5 μg/ml CpG 2006 (TIB MOLBIOL Syntheselabor, Berlin, Germany) and/or 2 μg/ml F(ab′)_2_ anti-human IgM/IgG (anti-BCR; Jackson ImmunoResearch, Suffolk, UK) for 48 hours at 37 °C in 5 % CO_2_ [4]. Because the anti-BCR can potentially bind to the crystallizable fragment (Fc) region of the whole anti-CD22 antibody epratuzumab, and because Sieger et al. [6] could show that epratuzumab and F(ab′)_2_ epratuzumab have the same effect on BCR signaling, the F(ab′)_2_ fragment of epratuzumab was used to study the effect of the anti-CD22 antibody on the cytokines production after TLR9 and/or BCR activation. For the cytokine analysis, supernatants from the B cell *in vitro* cultures were harvested and frozen at −70 °C. For the intracellular IL-10 staining, PBMCs stimulated *in vitro* were used. Unstimulated B cells were used as a negative control.

### Interleukin-10 staining for flow cytometry

Intracellular staining for IL-10 was performed on PBMCs after 2 days of *in vitro* culture. PBMCs were restimulated for the last 4 hours with 10 ng/ml phorbol myristate acetate and 700 ng/ml ionomycin, including 2 hours with 2 μg/ml brefeldin A (all from Sigma-Aldrich, Munich, Germany). PBMCs were stained first on ice for 10 minutes with antibodies against CD14-PacB (M5E2), CD3-PacB (UCHT1), CD27-FITC (L128), CD38-PerCP/Cy5.5 (HIT2) and CD20-PacO (H147) (all from BD Biosciences). After a washing step, PBMCs were incubated with 400 μl of BD FACS Permeabilizing Solution 2 (BD Biosciences) for 10 minutes at room temperature (RT). After another washing step, PBMCs were stained with anti-IL10-APC antibodies (JES3-9D7; Miltenyi Biotec) for 10 minutes at RT. Stained cells were analyzed by FC using a FACSCanto™ II flow cytometer and analyzed using FlowJo software. Unstimulated PBMCs with brefeldin A treatment were used as a control.

### Cytokine assay

Frozen supernatants of purified B cells stimulated *in vitro* were assessed for the cytokine concentrations of IL-1β, IL-4, lL-6, IL-10, IL-17A, IL-17F, IL-22, IL-23, IL-25, IL-31, IL-33, interferon-γ, sCD40L and TNF-α by using the Bio-Plex Pro™ Human Th17 Cytokine Panel (Bio-Rad Laboratories, Hercules, CA, USA) according to the manufacturer’s instructions. The assay sensitivity depends on the particular cytokines analyzed, ranging from 0.02 pg/ml for IL-1β to 2.13 pg/ml for IL-21. A significant and specific induction after anti-BCR and CpG stimulation above 20 pg/ml was observed only for the cytokines IL-6, TNF-α and IL-10. Therefore, the other cytokines were not considered in further analyses.

### Statistical analysis

The statistical analysis was performed with GraphPad Prism 5 software (GraphPad Software, San Diego, CA, USA). To compare HD and SLE groups, nonparametric Mann–Whitney *U* tests were applied. In comparative analyses of data with or without F(ab′)_2_ epratuzumab incubation, we applied the Wilcoxon signed-rank test test. *P* values <0.05 were considered to be statistically significant.

## Results

### Epratuzumab modulates the production of proinflammatory cytokines by activated B cells

**Fig. 1:**
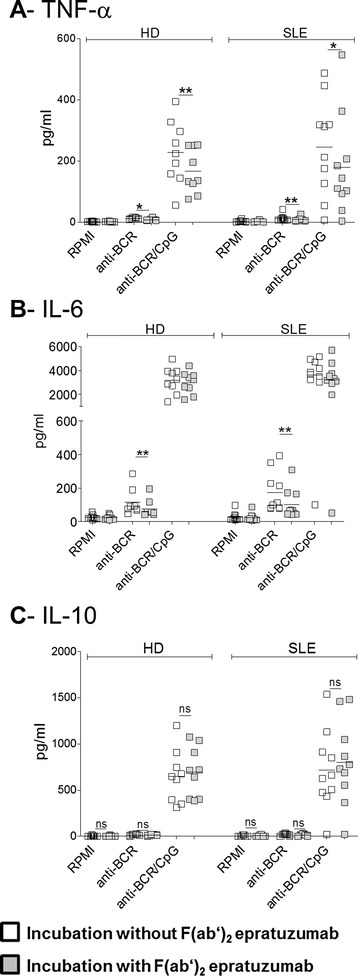
Targeting of CD22 by epratuzumab influences the secretion of the proinflammatory cytokines tumor necrosis factor (TNF)-α and interleukin (IL)-6, but not IL-10, by stimulated B cells. Peripheral blood B cells were purified from patients with systemic lupus erythematosus (SLE) (*left*) and healthy donors (HD) (*right*), pretreated with (*gray squares*) or without F(ab′)_2_ epratuzumab (*open squares*) and cultured with media alone (RPMI) or stimulated with anti–B cell receptor (anti-BCR) or anti-BCR + CpG. After 2 days of culture, the supernatants were harvested and tested for TNF-α, IL-6 and IL-10 protein production using a Bio-Plex Pro™ Human Th17 Cytokine Panel assay. Combined data from 13 patients with SLE and 9 HD are shown for TNF-α (**a**), IL-6 (**b**) and IL-10 (**c**) (Mann–Whitney *U* test; ns = not significant, *p < 0.05, **p < 0.01)

In initial studies, we evaluated whether the production of proinflammatory cytokines such as TNF-α and IL-6 by purified peripheral B cells from patients with SLE and HD differed upon activation by BCR alone or combined with TLR9 activation. The results showed that the most striking TNF-α induction in both HD and SLE B cells was for simultaneous activation of BCR and TLR9 in comparison with BCR activation alone (Fig. 1a). Notably, and consistent with previous data that epratuzumab partially inhibits BCR signaling [6], B cells pretreated with F(ab′)_2_ epratuzumab secreted significantly less TNF-α upon BCR cross-linking (HD: 11.7 ± 3.1 pg/ml, pretreated with F(ab′)_2_ epratuzumab 8.1 ± 3.8 pg/ml; *p* < 0.05; patients with SLE: 12 ± 9.3 pg/ml, pretreated with F(ab′)_2_ epratuzumab 8.1 ± 5.8 pg/ml; *p*<0.01) or upon combined stimulation (HD: 227.7 ± 103.8 pg/ml, pretreated with F(ab′)_2_ epratuzumab 167.0 ± 71.6 pg/ml;*p*<0.01;patients with SLE: 238.0 ± 147.1 pg/ml, pretreated with F(ab′)_2_ epratuzumab 181.5 ± 149.0 pg/ml; *p*<0.05). Thus, a clear inhibition was noted for TNF-α production by activated B cells from HD and patients with SLE under the influence of F(ab′)_2_ epratuzumab. This inhibition was seen after anti-BCR and anti-BCR + CpG activation. To exclude the possibility that F(ab′)_2_ epratuzumab has the ability to engage Fc receptors on B cells and would lead to an unspecific inhibition of TNF-α and IL-6 secretion after F(ab′)_2_ epratuzumab incubation, the binding of an F(ab′)_2_ epratuzumab isotype control was analyzed by FC and showed no binding to B cells or other cell subsets, including T cells, monocytes or natural killer (NK) cells (data not shown). These data are consistent with the notion that a specific binding of F(ab′)_2_ epratuzumab to CD22 on B cells was related to the significant reduction of TNF-α and IL-6 after BCR and anti-BCR + CpG activation.

In a subsequent analysis, IL-6 production by purified B cells from HD and patients with SLE was studied (Fig. 1b). As observed for TNF-α, combined stimulation induced a synergistic increase of IL-6 production by B cells from patients with SLE (3397 ± 1353 pg/ml) and comparably for B cells from HD (anti-BCR + CpG: 3199 ± 1097 pg/ml). The combined stimulation was again a more potent IL-6 inducer than BCR-cross-linking alone (anti-BCR; HD: 115.5 ± 80.4 pg/ml; SLE: 153.8 ± 109.8 pg/ml). F(ab′)_2_ epratuzumab was able to significantly reduce IL-6 production by anti-BCR alone F(ab′)_2_ epratuzumab was able to significantly reduce IL-6 production by anti-BCR alone (*p*<0.01) in HD and SLE cultures, whereas the inhibition by epratuzumab after combined stimulation was not statistically significant.

We then addressed the influence of epratuzumab on IL-10 production, a cytokine considered to be an immunoregulatory cytokine. Here the combined stimulation was found to be an important IL-10 inducer compared with anti-BCR alone (Fig. 1c). However, F(ab′)_2_ epratuzumab did not influence the secretion of IL-10 by B cells in any of the three stimulation conditions tested in both SLE and HD B cell cultures. There was a trend for B cells from patients with SLE to produce more IL-10 upon TLR9 and BCR cross-linking after pretreatment with F(ab′)_2_ epratuzumab (608 ± 420 pg/ml vs. 703 ± 452 pg/ml upon F(ab′)_2_ epratuzumab), which prompted further studies.

### Epratuzumab does not affect the frequency of interleukin-10–producing B cells

**Fig. 2:**
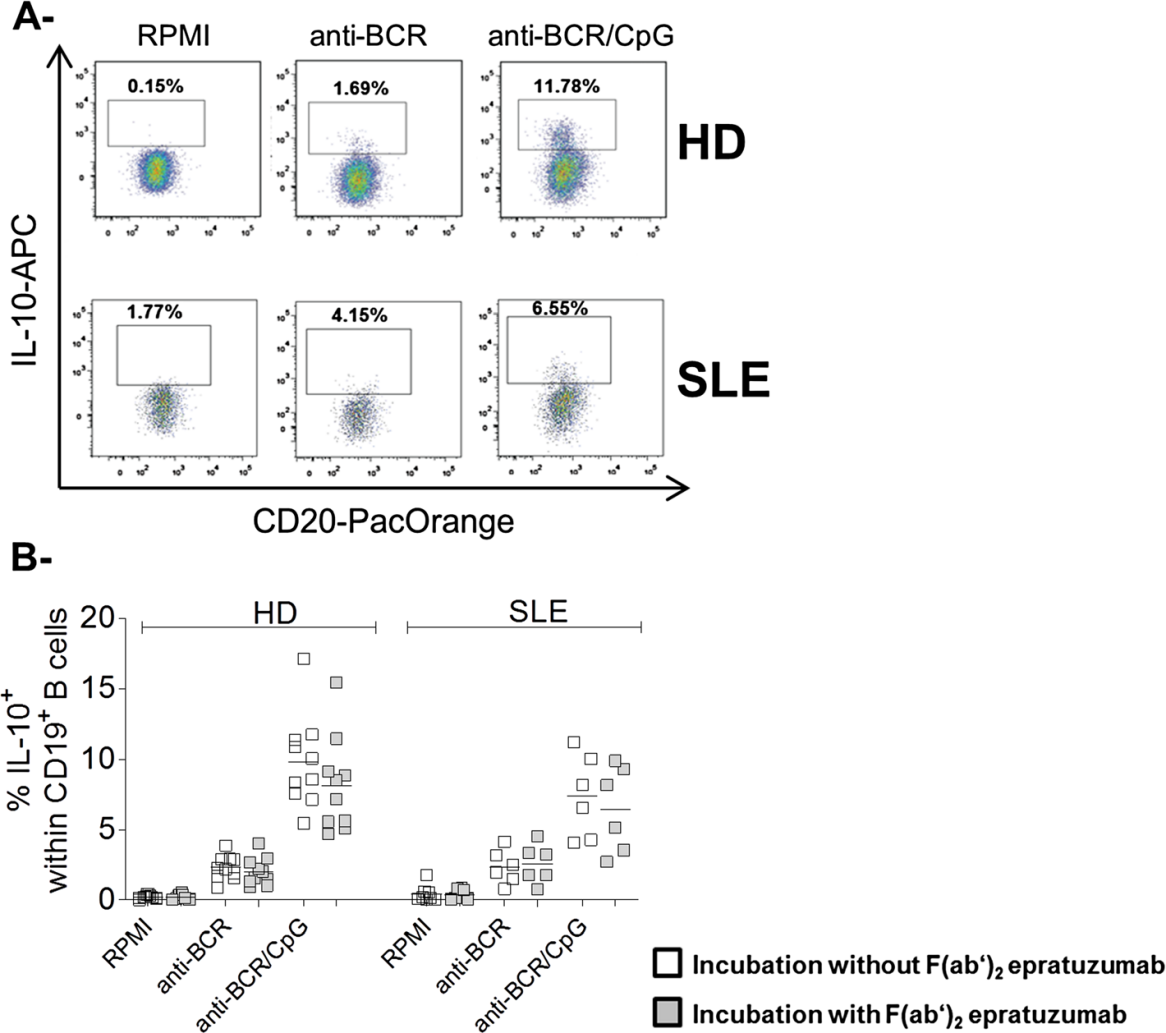
Epratuzumab does not influence the generation of intracellular interleukin (IL)-10-positive B cells. (**a**) Frequency of intracellular IL-10^+^ B cells after 2 days of peripheral blood mononuclear cell culture of representative healthy donors (HD) or patients with systemic lupus erythematosus, respectively, without stimulation (RPMI 1640 medium) or stimulated by anti–B cell receptor (anti-BCR) or anti-BCR + CpG using flow cytometry. (**b**) Comparison of the frequency of IL-10–producing B cells from 6 patients with SLE and 10 HD without (*open squares*) and with F(ab′)_2_ epratuzumab (gray squares) incubation in response to the indicated stimulations (Mann–Whitney *U* test)

IL-10–producing B cells in both humans and mice have been described as having regulatory functions and to be impaired in SLE [12–14]. Although epratuzumab did not substantially affect the secretion of IL-10 by B cells, the potential capacity of F(ab′)_2_ epratuzumab to generate IL-10–producing B cells *in vitro* was studied by direct identification of intracellular IL-10 using FC in B cells after 2 days of BCR cross-linking or combined BCR-TLR9 stimulation (Fig. 2a). Simultaneous BCR-TLR9 activation induced the highest frequency of IL-10–producing B cells in HD (9.8 ± 3.3 %), as well as in patients with SLE (7.4 ± 3.0 %), which was consistent with the detection of secreted IL-10 (Fig. 1c). There was no influence of F(ab′)_2_ epratuzumab on the generation of IL-10–producing B cells from either patients with SLE or HD, independent of the stimulation conditions applied (Fig. 2b).

### Epratuzumab increases the ratio of interleukin-10 to proinflammatory cytokines produced by B cells from patients with systemic lupus erythematosus

**Fig. 3:**
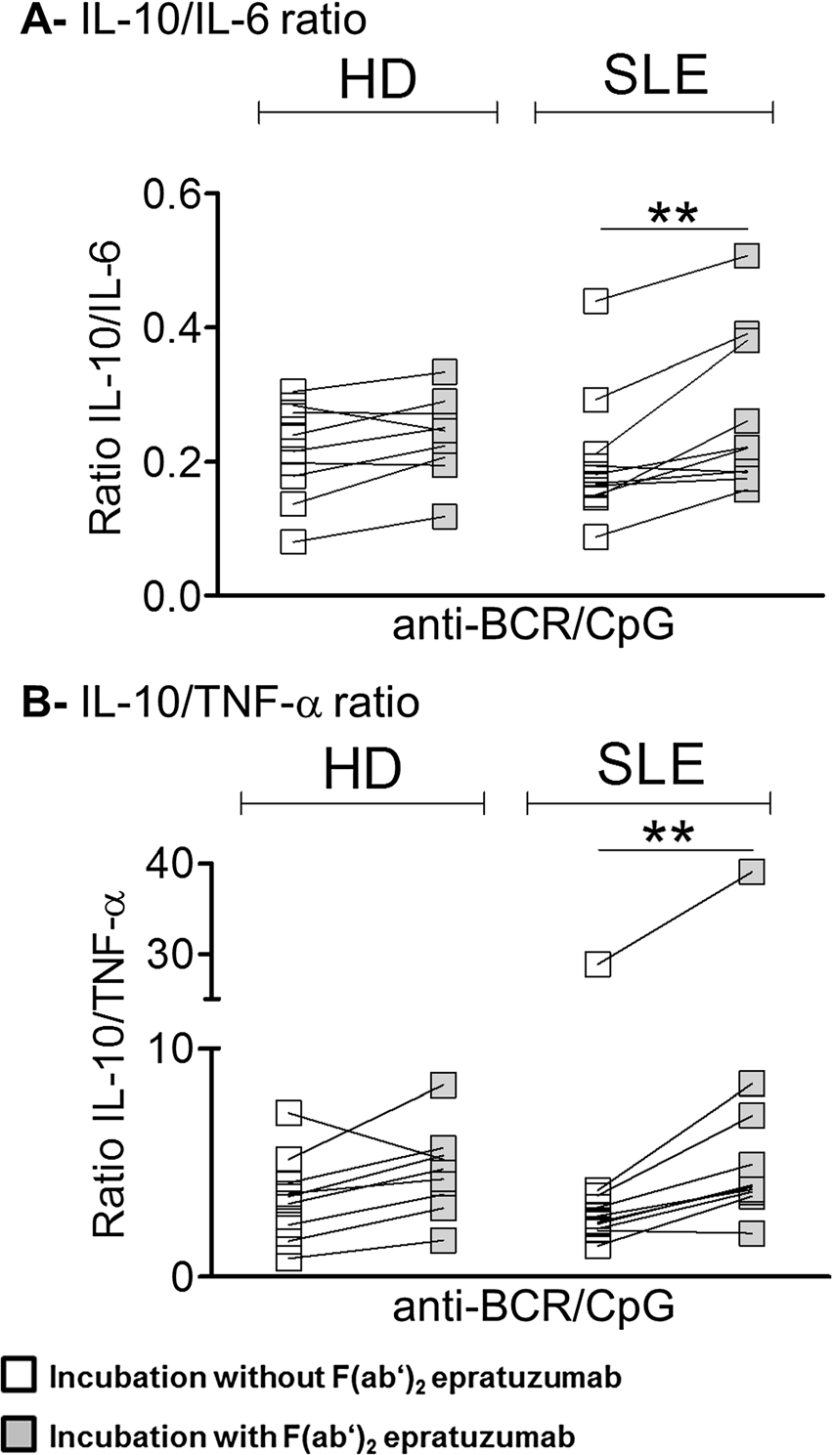
Epratuzumab influences the balance between interleukin (IL)-10 and the proinflammatory cytokines tumor necrosis factor (TNF)-α and IL-6 secreted by B cells activated by anti–B cell receptor (anti-BCR) + CpG in patients with systemic lupus erythematosus (SLE). The balance between IL-10 and proinflammatory cytokines (IL-6 and TNF-α) produced by B cells from healthy donors (HD) and patients with SLE was evaluated based on the ratio of IL-10 to IL-6 (**a**) or IL-10 to TNF-α (**b**) (Mann–Whitney *U* test; **p < 0.01)

To assess whether epratuzumab influences the balance between IL-10 and the two tested proinflammatory cytokines, the ratios of IL-10 to IL-6 or TNF-α were analyzed for anti-BCR + CpG stimulation in the individual donors (Fig. 3).

As a result, the ratio of IL-10 to IL-6 upon anti-BCR + CpG stimulation was lower in patients with SLE (0.18 ± 0.1) than in HD (0.21 ± 0.07), but this difference was not significant. F(ab′)_2_ epratuzumab influenced the ratio of IL-10 to IL-6 in patients with SLE (0.24 ± 0.12) and in HD (0.24 ± 0.06), indicating a change in cytokine balance in favor of IL-10 as a regulatory cytokine. This difference between the conditions with or without F(ab′)_2_ epratuzumab was statistically significant for B cells from patients with SLE (*p*<0.01) (Fig. 3a). Similar results were obtained for the ratio of IL-10 to TNF-α, whereby F(ab′)_2_ epratuzumab significantly influenced the cytokine balance toward IL-10 in B cells from patients with SLE (*p* < 0.01) (Fig. 3b). Overall, the ratios of IL-10 to either IL-6 or TNF-α were not related to the underlying disease activity (according to SLEDAI score) (data not shown).

## Discussion

In this study, we assessed the effect of the anti-CD22 mAb epratuzumab on the production of specific cytokines upon BCR and combined BCR and TLR9 B cell stimulation *in vitro*. As a key result, cytokines produced following BCR activation were TNF-α and IL-6, and both were substantially reduced upon epratuzumab exposure. Interestingly, the secretion of the immunoregulatory cytokine IL-10 in culture supernatants and intracellular IL-10 production were largely dependent on TLR9 activation by CpG in both HD and patients with SLE, and this production was not influenced by epratuzumab. Of particular note, immunoregulatory cytokines appear to be less dependent on BCR activation, but they have been reported to be intimately involved in regulatory circuits of IL-10 and IL-35 production [15].

This study confirms that B cells can be activated by different pathways, resulting in a distinct modulation of the production of certain cytokines. All cytokines studied were produced at the highest level in the combined stimulation settings, but it was clear that IL-10 induction was independent of BCR activation. Whereas enhanced IL-10 production by *cis* signaling of Siglec family members has been reported for DCs and macrophages [7], the role of BCR and TLR9 activation on B cells from controls and patients has not been reported before. Moreover, reports of the production of IL-10 by B cells stimulated with different stimuli have been controversial. Several studies showed an inhibitory effect of anti-IgM stimulation on IL-10 production by B cells [13], whereas other studies [16], consistent with our study, showed a synergistic effect of anti-BCR or anti-BCR + CpG to produce IL-10. A principal difference that may explain the different results between these studies is the different isotypes used to stimulate the BCR, such as IgM only; IgM and IgG; or IgM, IgG and IgA. Given the inherent regulatory function of CD22 for BCR activation, the data we present also support the notion that epratuzumab has a striking effect on BCR-dependent cytokine production (IL-6 and TNF-α) but leaves the TLR9-dependent axis largely intact. This regulatory dichotomy appears to be important to explain the distinct effects when targeting CD22 that is closely linked to downstream functions of BCR activation.

It should be emphasized that the influence of epratuzumab on IL-6, TNF-α and IL-10 production was comparable between SLE B cells and controls, but that the ratios of IL-10 to TNF-α and IL-10 to IL-6 were substantially different in SLE upon BCR-TLR9 stimulation. Although a potential influence of non-B cells contributing to the *in vitro* cytokine production cannot be absolutely excluded, the mean B cell purity of 98.5 ± 2.2 % with only two samples with 8.2 % and 5.6 % non-B cells did not likely result in a substantial difference in the influence of the mAb against CD22, because this binding is very B cell–specific. Moreover, F(ab′)_2_ epratuzumab has been chosen to ensure that the observed effects can be attributed to a specific CD22 binding and to avoid potential Fc receptor effects, especially on monocytes, validated by FC using an F(ab′)_2_ isotype control. However, current studies in our laboratory address the comprehensive impact of intact epratuzumab on PBMC cultures, including indirect effects on T cells and monocytes. Because we used negatively selected B cells to avoid any additional preactivation of B cells by other purification methods, we believe that the ratios reflect the situation in individual patients. However, the influence of the mAb in treated patients needs to be fully explored.

## Conclusions

Epratuzumab targets the BCR coreceptor CD22 and was found to substantially inhibit the *in vitro* production of proinflammatory IL-6 and TNF-α by B cells, likely related to their close dependence on BCR signaling [6]. In contrast, IL-10 production, reportedly less dependent on BCR activation, was not substantially influenced by epratuzumab. These data suggest that epratuzumab alters the balance between the production of proinflammatory cytokines (TNF-α, IL-6) and the regulatory cytokine IL-10 as another B cell effector mechanism. The findings provide further evidence that epratuzumab can affect downstream BCR functions such as particular cytokines produced by B cells.

## Abbreviations

BCR: B cell receptor
DAPI: 4′,6-diamidino-2-phenylindole
DC: dendritic cell
Fc: crystallizable fragment
FC: flow cytometry
HD: healthy donors
Ig: Immunoglobulin
IL: interleukin
mAb: monoclonal antibody
NK: natural killer
PBMC: peripheral blood mononuclear cell
RT: room temperature
Siglec: sialic acid–binding immunoglobulin-type lectin
SLE: systemic lupus erythematosus
SLEDAI: Systemic Lupus Erythematosus Disease Activity Index
TLR: Toll-like receptor
TNF: tumor necrosis factor
